# An *In Vitro* Model of the Glomerular Capillary Wall Using Electrospun Collagen Nanofibres in a Bioartificial Composite Basement Membrane

**DOI:** 10.1371/journal.pone.0020802

**Published:** 2011-06-24

**Authors:** Sadie C. Slater, Vince Beachley, Thomas Hayes, Daming Zhang, Gavin I. Welsh, Moin A. Saleem, Peter W. Mathieson, Xuejun Wen, Bo Su, Simon C. Satchell

**Affiliations:** 1 Academic Renal Unit, University of Bristol, Bristol, United Kingdom; 2 Department of Bioengineering, Clemson University, Charleston, South Carolina, United States of America; 3 Department of Oral and Dental Science, University of Bristol, Bristol, United Kingdom; Massachusetts Institute of Technology, United States of America

## Abstract

The filtering unit of the kidney, the glomerulus, contains capillaries whose walls function as a biological sieve, the glomerular filtration barrier. This comprises layers of two specialised cells, glomerular endothelial cells (GEnC) and podocytes, separated by a basement membrane. Glomerular filtration barrier function, and dysfunction in disease, remains incompletely understood, partly due to difficulties in studying the relevant cell types *in vitro*. We have addressed this by generation of unique conditionally immortalised human GEnC and podocytes. However, because the glomerular filtration barrier functions as a whole, it is necessary to develop three dimensional co-culture models to maximise the benefit of the availability of these cells. Here we have developed the first two tri-layer models of the glomerular capillary wall. The first is based on tissue culture inserts and provides evidence of cell-cell interaction via soluble mediators. In the second model the synthetic support of the tissue culture insert is replaced with a novel composite bioartificial membrane. This consists of a nanofibre membrane containing collagen I, electrospun directly onto a micro-photoelectroformed fine nickel supporting mesh. GEnC and podocytes grew in monolayers on either side of the insert support or the novel membrane to form a tri-layer model recapitulating the human glomerular capillary *in vitro*. These models will advance the study of both the physiology of normal glomerular filtration and of its disruption in glomerular disease.

## Introduction

The glomerular capillary wall consists of three specialised layers: glomerular endothelial cells (GEnC) on the inside of the capillary, a glomerular basement membrane (GBM) and a layer of podocytes (glomerular epithelial cells) on the outside. The cell layers are separated by a relatively thick basement membrane (300–350 nm), the GBM, a specialised form of extracellular matrix (ECM). It is predominantly composed of particular isoforms of matrix proteins: collagen type IV α3, α4 and α5 chains, and laminin-11 [1], [2]. The three layers of this system function as a whole, the glomerular filtration barrier (GFB), to selectively filter the blood [3]. Paracrine communication via soluble mediators between GEnC and podocytes is important in maintenance of the structure and function of the GFB. For example we and others have shown that podocytes secrete the endothelial factors VEGF-A, VEGF-C and angiopoietin 1, whilst GEnC express the cognate receptors for these mediators [4], [5], [6], [7]. Podocyte-specific alterations of VEGF-A or angiopoietin expression results in GFB dysfunction including GEnC abnormalities and proteinuria [8], [9].

Despite the definition of this structure of the glomerular capillary wall by electron microscopy as far back as 1957 [10], understanding of its filtration function remains incomplete. *In vitro* work historically has been limited by difficulties with culturing cells of the filtration barrier. Recently, improved culture techniques, and in particular generation of unique conditionally immortalised cell lines of both human GEnC and podocytes in our laboratory, have enabled detailed study of these cells *in vitro* [11], [12]. However, because the components of the glomerular capillary wall function as an inter-related whole, there is a need to develop three-dimensional co-culture models to maximise the relevance of *in vitro* studies to the intact glomerulus. Ideally such a model would be comprised of a layer of GEnC and a layer of podocytes, separated by a biological membrane acting as the GBM. This membrane should be thin, permeable, able to support the structure, biological, biodegradable, composed of proteins found in the GBM and accessible on both sides (to enable measurement of fluid and molecular movement across the structure).

Co-culture techniques have been successfully used to generate functional *in vitro* models of other tissues, such as the blood-brain barrier [13], skin [14] and the lung alveolar capillary barrier [15]. One of the main challenges in co-culture models is generation of an ECM or basement membrane most appropriate for the tissue in question. In some cases basement membrane has been represented by the synthetic porous support of a tissue culture insert [13], [15], [16], [17]. However basement membrane is more than just a scaffold providing support: it also regulates cellular behaviour by influencing proliferation, survival, shape, migration and differentiation [18]. Therefore ideally an artificial GBM should closely mimic the structure and function of the native matrix proteins. Collagen type I has a high degree of biocompatibility and is biodegradable and has therefore been used to mimic the basement membrane in various models, for example of the blood brain barrier [19]. Other biocompatible gels have been used in similar co-culture models, such as self-assembled peptide hydrogel [20] and Matrigel [21]. Usually these components are prepared as thick gels and at least in this form they are not suitable to represent the GBM. Electrospinning has recently come to the fore as a relatively simple technique which can produce fibres on a nanometre scale in random or aligned patterns forming a structure that can mimic the ECM [22]. Biocompatible and biodegradable nanofibres of various synthetic (such as polyglycolic acid [23] and polycaprolactone, PCL [14]) or naturally occurring (such as collagen type I [22] and chitosan [24]) polymers have been produced, the later being of most relevance to the synthesis of a GBM-like structure. A number of different cell types have been successfully cultured onto collagen type I electrospun membranes including coronary endothelial cells [25], fibroblasts [26] and keratinocytes [27], therefore it is used extensively as an ECM component in tissue engineering. The incorporation of PCL in electrospun collagen nanofibres is attractive to increase their tensile strength whilst also being biocompatible and biodegradable [14], [28].

Previously, a few co-culture models have examined interactions between glomerular cells [17], [29], [30], [31], but no model of the glomerular capillary wall including glomerular endothelial cells, basement membrane and podocytes yet exists. Here we describe the sequential construction of two *in vitro* models of the glomerular capillary wall fulfilling these desirable attributes. We tested two hypotheses, firstly that it would be possible to established a tri-layer model based on tissue culture inserts, with a porous artificial membrane acting as the GBM. Secondly, we hypothesised that we could develop a composite bioartificial membrane consisting of electrospun collagen type I and a supporting micro photo-electroformed (micro-PEF) nickel mesh to replace the porous membrane in the first model.

## Results

### Co-culture of cells on insert membranes

**Figure 1:**
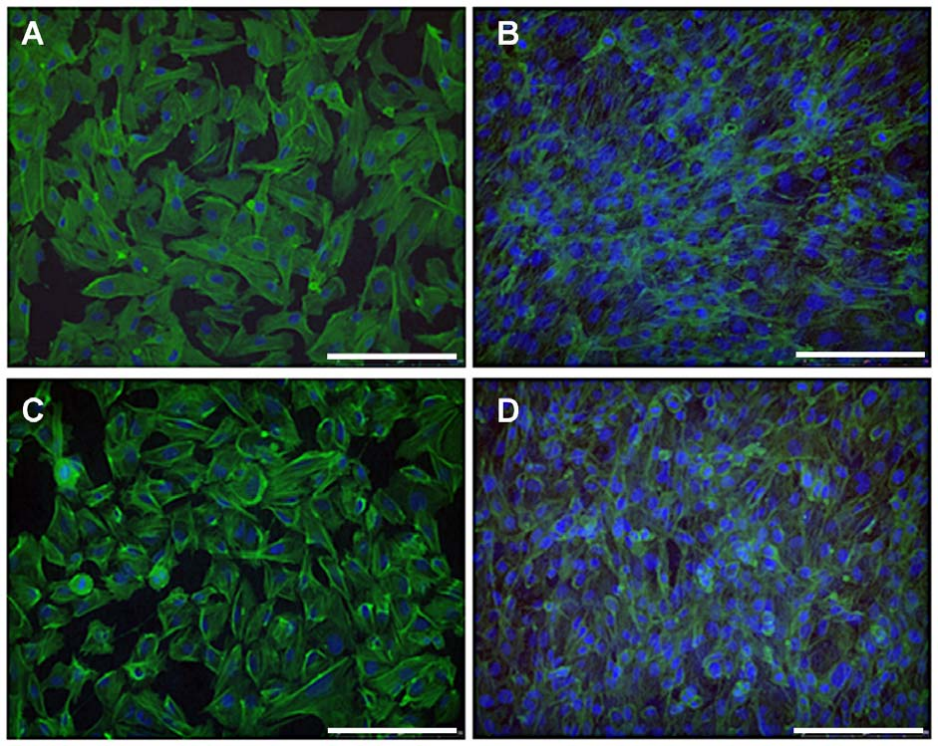
IF staining for actin (green) and nuclei (blue) showing GEnC and podocyte monolayer formation in tissue culture inserts after a week at 33°C. (A) GEnC cultured alone in a single monolayer. (B) GEnC when podocytes co-cultured on the opposite side of the insert. (C) Podocytes cultured alone in a single monolayer. (D) Podocytes when GEnC co-cultured on the opposite side of the insert. The number of cells, and the confluence of the monolayer, appears to increase when the cells are co-cultured. Scale bar: 250 µm.

**Figure 2:**
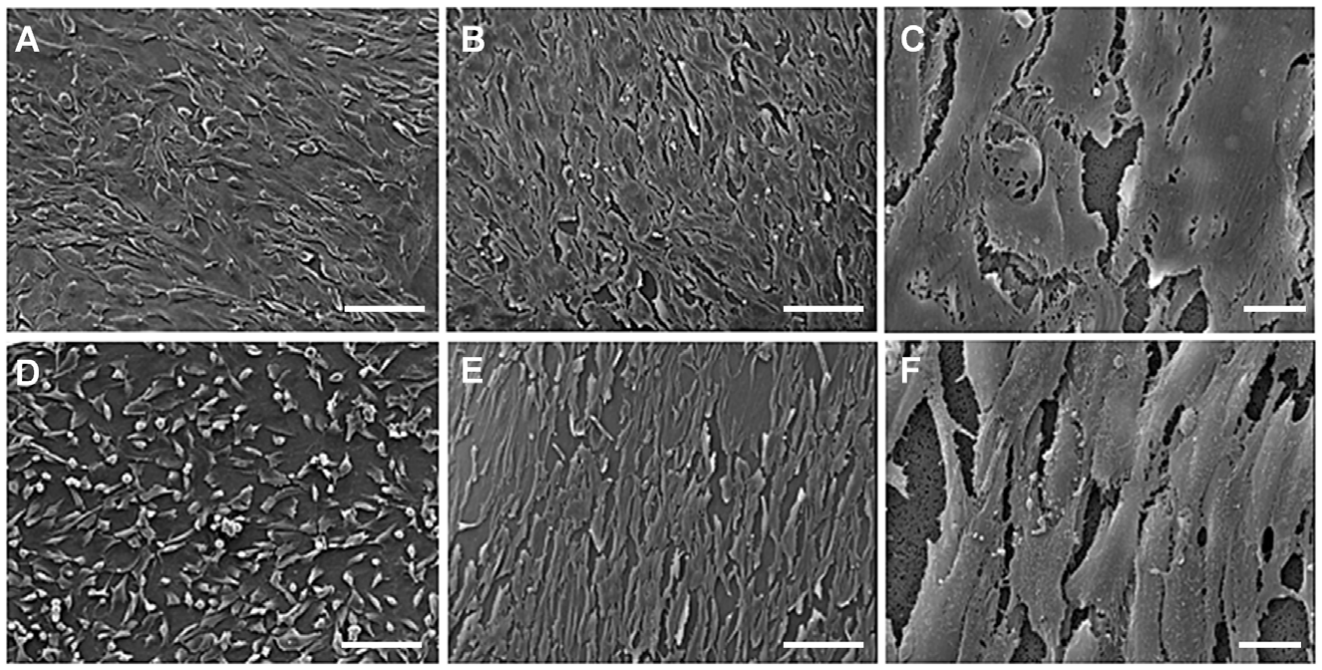
Scanning EM demonstrating morphology of GEnC (A–C) and podocytes (D–F) when co-cultured on opposite sides of a tissue culture insert porous support. Cells were cultured for 1 week at 33°C (A and D) or a week at 33°C followed by a week at 37°C (B,C,E and F). Scale bars 100 µm (A,B,D and E) or 10 µm (C and F). Images acquired at accelerating voltage of 15 kV. GEnC and podocytes each form a monolayers under co-culture conditions.

GEnC and podocytes grew in uniform layers on either side of the polycarbonate support to form a representative model of the glomerular capillary wall (Fig. 1). The density of both cell types appeared to increase when co-cultured opposite the other cell type. Similar results were obtained at 33°C (Fig. 1) and 37°C (data not shown). Scanning EM further confirmed growth of GEnC and podocytes on the polycarbonate support. At both 33°C (Fig. 2A) and 37°C (Fig. 2B and C) GEnC grew in uniform layers when co-cultured with podocytes. Podocytes in co-culture with GEnC at 33°C (Fig. 2D) displayed a more rounded morphology, however when co-cultured at 37°C these cells became flatter and formed a single layer (Fig. 2E and F).

**Figure 3:**
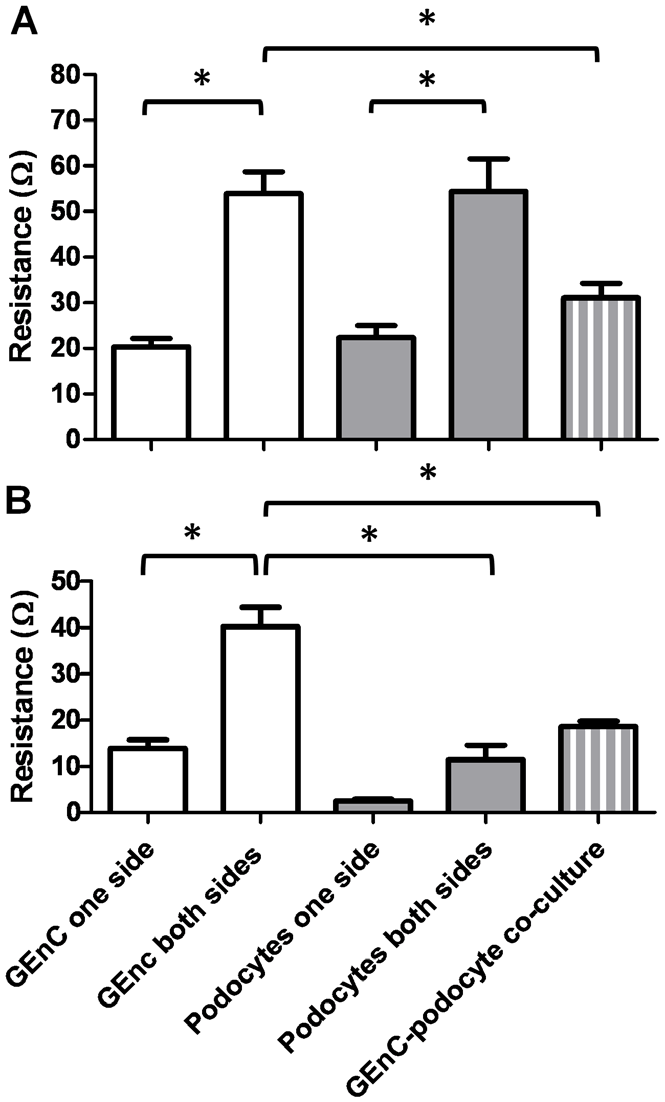
Barrier properties of the cells cultured in tissue culture inserts in single layers and co-culture was measured by TEER. Cells were cultured for 1 week at 33°C (A) or a week at 33°C followed by a week at 37°C (B). Bars show mean +/− SEM, n = 20 (mean result of 4 experiments, n = 5 in each). At both temperatures resistance increased when cells in co-culture were compared to cells in a single layer. * p<0.05 by ANOVA with Bonferroni post hoc tests.

The barrier properties of this co-culture model were assessed by measuring trans-endothelial electrical resistance (TEER). TEER is inversely related to the fractional area of pathways across a cell monolayer open to water and small molecules and as such can be used an index of permeability, a higher TEER corresponding to reduced permeability. At 33°C (Fig. 3A) resistance of inserts with GEnC layers both sides was significantly higher than those with a single layer of GEnC (20+/−1.7 Ω compared to 53+/−4.7 Ω, p<0.005). Likewise for podocyte single and dual layers (22+/−2.8 Ω compared to 54+/−7 Ω, p<0.005). There was no significant difference in resistance when dual layers of GEnC and podocytes were compared. However in comparison to dual layers of GEnC or podocytes, the GEnC and podocyte mixed cell type co-culture had a significantly lower resistance (31+/−3 Ω, p<0.003 and p<0.002 respectively). At 37°C (Fig. 3B) dual GEnC layers again showed more than a doubling of resistance compared to a single layer of GEnC (13.8+/−1.8 Ω compared to 40.2+/−4 Ω, p<0.001). Dual layers of podocytes showed an almost four-fold increase of resistance values compared to podocyte monolayers (2.4+/−0.4 Ω compared to 11.4+/−3 Ω), however this change was not significant. When these resistances were compared to the GEnC and podocyte co-culture resistance (18.6+/−1 Ω), the mixed cell-type co-culture had a significantly lower resistance than the GEnC dual layers (p<0.005), but no difference when compared to the podocyte dual layers. Also, podocyte dual layers had a significantly lower resistance than GEnC co-culture (p<0.005). GEnC resistances in general are comparable between 33 and 37°C [11], [12] whereas podocyte resistances are lower at 37°C as we have previously observed [32].

### GEnC behaviour on biological matrices

**Figure 4:**
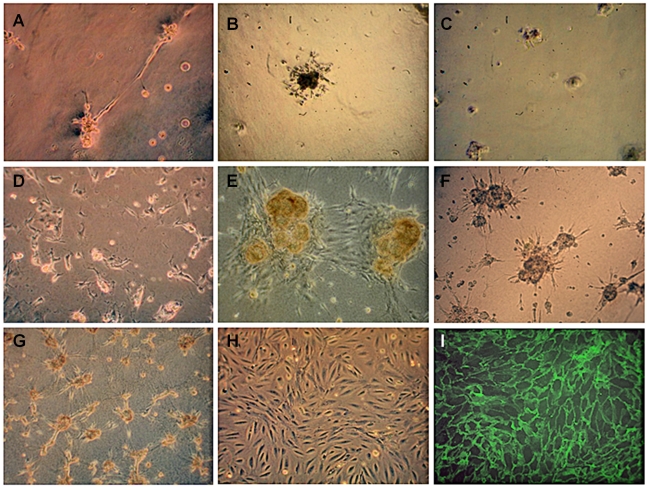
Appearance of GEnC cultured for 1 week at 33°C on various matrices to identify a suitable matrix to support growth of GEnC in a uniform layer by light (A–H) or immunofluorescence (I) microscopy. (A) GEnC formed capillary tube-like structures on reduced growth factor (RGF) Matrigel, (B) clumps on RGF Matrigel coated with fibronectin or (C) gelatin, (D) rounded up on peptide hydrogel, (E) formed clumps on collagen type I and on (F) collagen type I coated with 20 µg/ml fibronectin or (G) gelatin. (H) GEnC formed a confluent monolayer on tissue culture plastic and on (I) Cellagen (visualised using IF staining for VE-cadherin, as Cellagen is opaque). All cells were seeded at the same density. Original magnification ×10.

GEnC cultured on reduced growth factor (RGF) Matrigel formed tubes, whilst on RGF Matrigel coated with fibronectin or gelatin, clumps of cells were formed (Fig. 4A–C). GEnC also failed to form a monolayer on a peptide hydrogel, collagen type I gel, and collagen type I gels coated with fibronectin or gelatin (Fig. 4D–G). GEnC only formed a confluent monolayer similar to that seen on tissue culture plastic (Fig. 4H) when seeded onto Cellagen (Fig. 4I).

### Electrospinning of collagen/PCL

Cellagen is available only as a thick (35 µm) layer which is impermeable to molecules larger than 3000–4000 Daltons and therefore unsuitable for a model of the GFB. However, it did serve to demonstrate that GEnC will form a confluent monolayer on a collagen membrane provided it is in a suitable form. As an alternative we electrospun a solution of type I collagen and PCL to form a nanofibre membrane, which was stabilised by cross-linking. Scanning electron microscopy demonstrated the fibrous nature of the membranes produced (images of membrane alone not shown, see below for membrane electrospun onto nickel mesh).

### Cell monolayer formation on electrospun membrane

**Figure 5:**
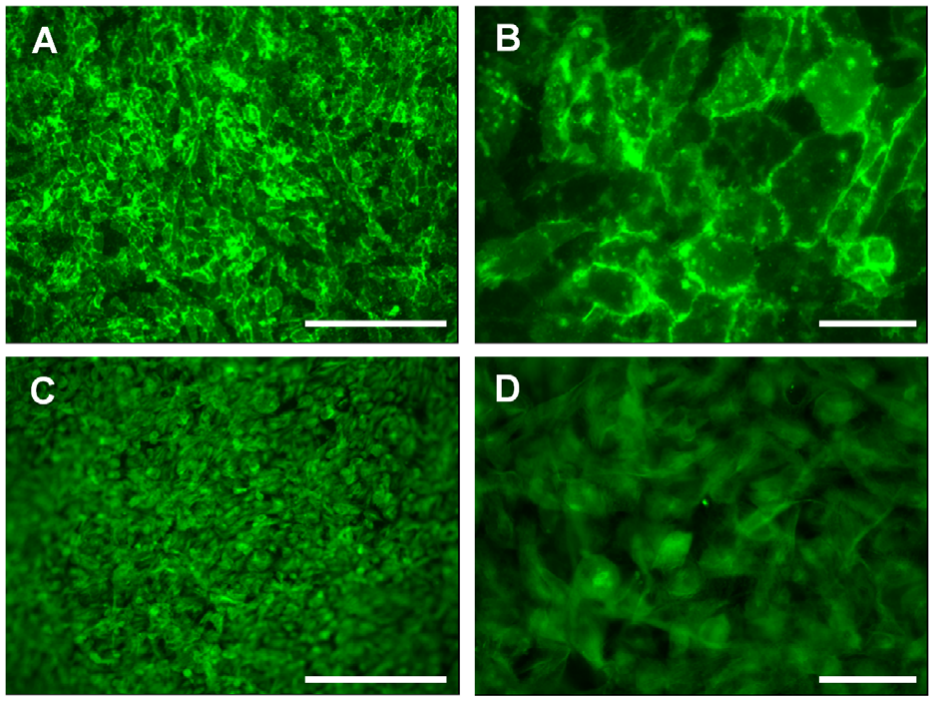
Immunofluorescence staining of (A and B) GEnC and (C and D) podocytes demonstrating monolayer formation when cells are cultured on collagen/PCL electrospun membranes for 1 week at 33°C. GEnC are stained for PECAM-1 and podocytes for podocin. Scale bars bar 250 µM (A and C) and 50 µm (B and D).

IF staining using cell-specific antibodies demonstrate that GEnC and podocytes formed confluent monolayers on the collagen/PCL membrane when incubated at 33°C for one week (Fig. 5).

**Figure 6:**
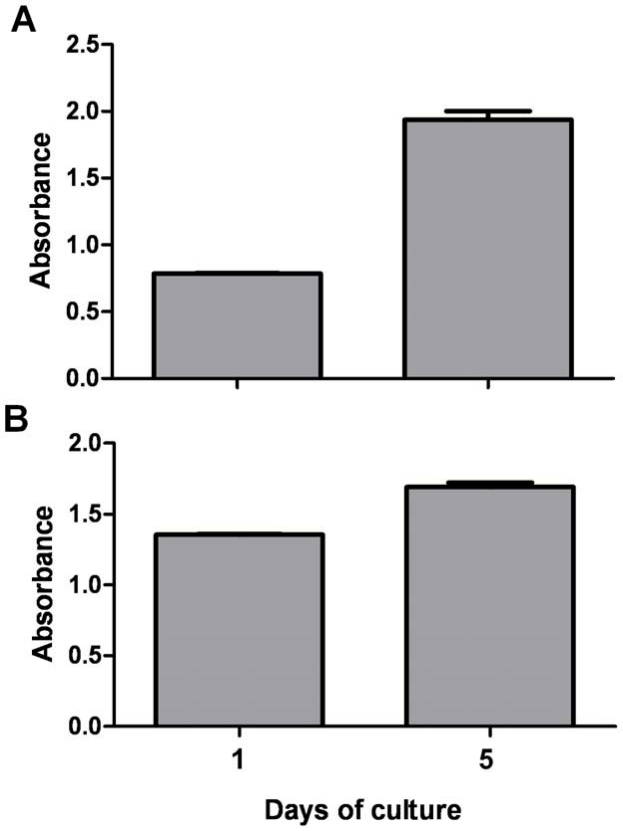
A WST-1 assay demonstrating proliferation of (A) GEnC and (B) podocytes on collagen/PCL electrospun membranes at 33°C. Cell number is proportional to absorbance and increases over time in each a case (p<0.05, n = 3).

GEnC and podocyte growth on the collagen/PCL membrane was studied using a WST-1 assay performed 1 and 5 days after seeding (Fig. 6). There was a significant increase in viable cells for both cell types (GEnC p<0.003, podocytes p<0.01) over this time period confirming their proliferation, in addition to attachment, on the collagen/PCL membrane.

### Formation of the composite bioartificial GBM

**Figure 7:**
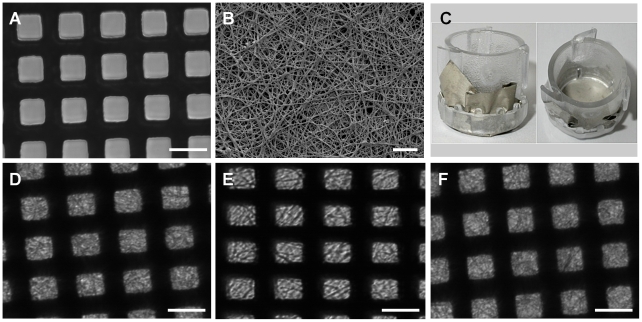
Illustrative images of the micro-PEF nickel mesh and the mesh coated with electrospun nanofibres to form a bioartificial composite membrane. (A) Light microscopy of micro-PEF nickel mesh (scale bar 25 µm). The black lines are the bars of the mesh, white squares are the open area. (B) Scanning EM of the micro-PEF nickel mesh coated with electrospun collagen/PCL nanofibres, scale bar 10 µm. (C) Collagen I/PCL/nickel mesh bioartificial composite membrane secured in 10 mm diameter Cell Crowns. (D,E and F) Light microscopy of the collagen/PCL/mesh composite after 1, 3 and 5 days incubation in tissue culture media at 33°C (scale bar 25 µm).

A micro-PEF nickel mesh (Fig. 7A) was identified as a suitable structural support in view of its physical characteristics and lack of toxicity to cells as assessed by adherence and growth directly on this mesh (data not shown). Next, the collagen/PCL solution was successfully electrospun directly onto this mesh (Fig. 7B) and the resulting biocomposite membrane was then placed in a Cell Crown to form a culture insert (Fig. 7C). Incubation of the membrane/mesh composite in cell culture media at 33°C for 1, 3 and 5 days did not affect membrane fibre appearance over time (Fig. 7C, D and E).

### Characterisation of cell growth on the composite bioartificial GBM

**Figure 8:**
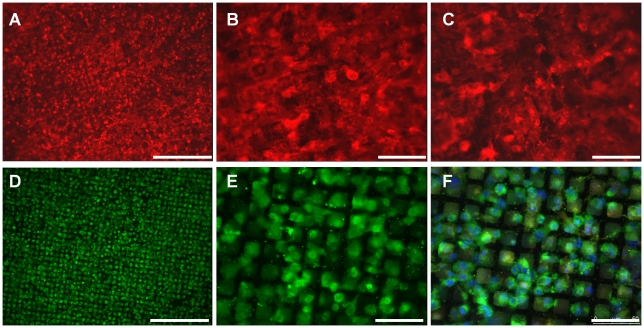
Immunofluorescence demonstrating appearance of GEnC (A–C) and podocyte (D–F) cell layers after one week at 33°C on the collagen/PCL/mesh bioartificial composite membrane. PECAM-1 on GEnC labelled red, podocin on podocytes green and nuclei blue (F only). (A and B) GEnC cultured in a single layer or (C) with podocytes co-cultured on the opposite side of the membrane. (D and E) podocytes cultured in a single layer or (C) with GEnC co-cultured on the opposite side of the membrane. Scale bars 250 µm (A and D) and 50 µm (B,C, E and F).

**Figure 9:**
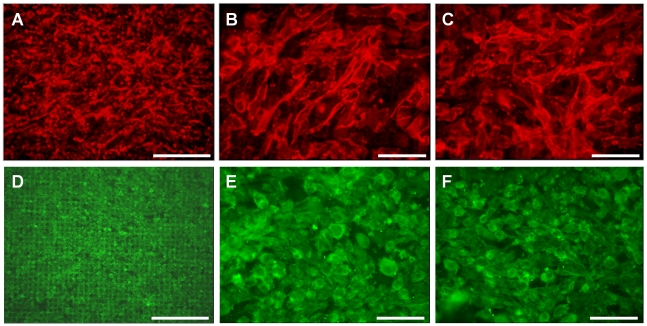
Immunofluorescence demonstrating appearance of GEnC (A–C) and podocyte (D–F) cell layers after one week at 33°C and one week at 37°C on the collagen/PCL/mesh bioartificial composite membrane. PECAM-1 on GEnC labelled red and podocin on podocytes green. (A and B) GEnC cultured in a single layer or (C) with podocytes co-cultured on the opposite side of the membrane. (D and E) podocytes cultured in a single layer or (C) with GEnC co-cultured on the opposite side of the membrane. Scale bars 250 µm (A and D) and 50 µm (B, C, E and F).

GEnC and podocytes were cultured on the electrospun collagen/PCL nickel mesh composite in single layers and in co-culture. After one week at 33°C GEnC formed a confluent monolayer on the collagen/PCL composite (Fig. 8A and B), whilst podocytes formed a sub-confluent layer (Fig. 8D and E). When the cells were grown in co-culture, GEnC still formed a monolayer and no podocin staining could be seen on the endothelial side (Fig. 8C). Podocytes again formed a sub-confluent monolayer and PECAM-1 staining on GEnC on the opposite side of the bioartificial membrane could be faintly observed (Fig. 8F). When the cells were cultured at 37°C, both GEnC and podocytes formed a confluent monolayer when grow in either a single layer (Fig. 9A–E) or co-culture (Fig. 9C and F). There was no apparent difference in the expression level or distribution of PECAM-1 or podocin between cells cultured on inserts and the bioartificial membrane or between cells in monoculture and in coculture.

**Figure 10:**
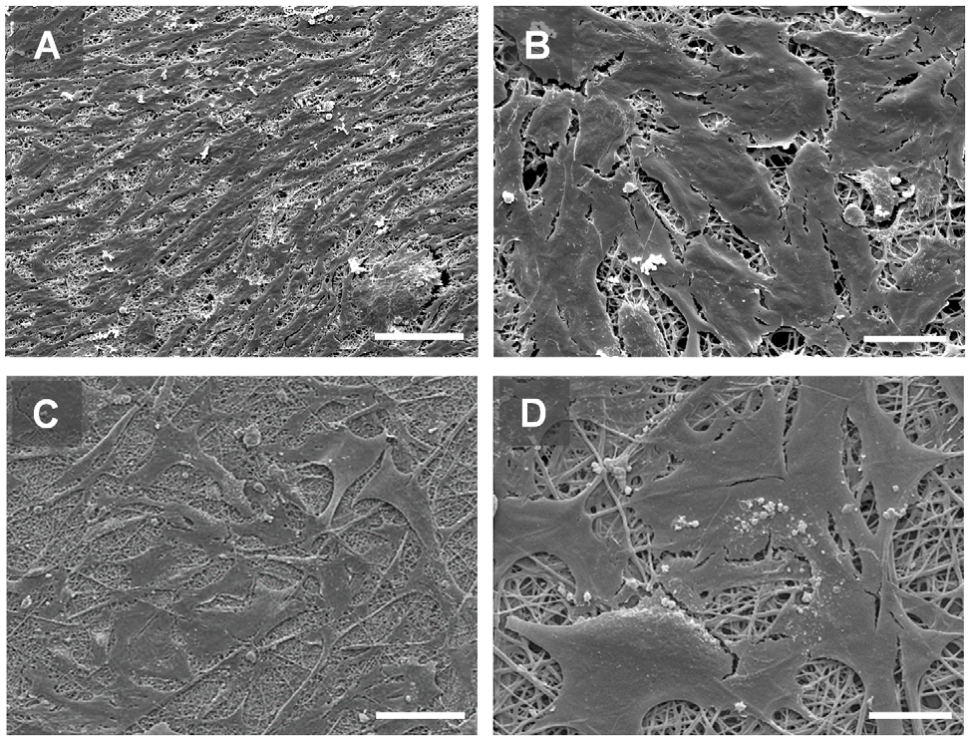
Scanning EM demonstrating morphology of GEnC (A and B) and podocytes (C and D) co-cultured on opposite sides of the collagen/PCL/mesh bioartificial composite membrane. Scale bars 100 µm (A and C) and 20 µm (B and D). GEnC form a uniform monolayer, comparable with those grown in monoculture or co-culture with podocytes on porous supports in tissue culture inserts (Fig. 2), whilst podocytes are less densely packed.

Scanning electron microscopy of GEnC and podocytes co-cultured on opposite sides of the collagen/PCL/mesh composite demonstrated that GEnC grow in a uniform monolayer (Fig. 10A and B), similar to those seen when these cells were co-cultured on the polycarbonate insert (Fig. 2A–C), whilst podocytes are less densely packed.

## Discussion

We have previously shown that tissue culture inserts can be used to culture human GEnC and podocyte monolayers [6], [7], [32]. Here we show for the first time that these cultures can effectively be combined either side of the porous support to form a tri-layer model. Cell morphology was preserved when grown on the underside of the support and when in the presence of the other cell type on the other side of the support (Figs. 1 & 2).

For both cell types in monoculture the TEER measurements (Fig. 3) were in agreement with previous values [12], [32]. The resistance of dual layers of the same cell type at either 33°C or 37°C, was at least double that a single monolayer of either GEnC or podocytes as expected. When GEnC and podocytes were co-cultured at 33°C on opposite sides of the insert, the combined resistance was higher than that of a single layer of either GEnC or podocytes. However, from the resistances of dual layers of the same cell type, the resistance would be expected to be at least the sum of the resistances of single GEnC and podocyte layers. The fact that the resistance was significantly lower than this suggests that one of the cell layers has reduced the resistance of the other, presumably via soluble mediators. A possible candidate for this is VEGF, as this growth factor is produced by podocytes [4], GEnC have receptors for VEGF [5], and VEGF is known to affect GEnC resistance [7]. For differentiated cells (at 37°C) in co-culture the combined resistance of layers of GEnC and podocytes was greater than the ‘expected resistance’. Therefore this does not suggest the same effect of cell-cell communication as seen in undifferentiated cells. The fact that there is a splicing switch in differentiation of podocytes to produce VEGF-A_165_b [33], which does not reduce GEnC resistance, could potentially explain this observation [34]. These results confirm that tri-layer models of the GFB can successfully be established *in vitro* and provide evidence that the cell-cell communication which occurs *in vivo* can be studied *in vitro*.

Given the dissimilarity between polycarbonate porous supports in inserts and the GBM *in vivo*, we sought to identify a suitable preparation of ECM components with which to replace the porous support as a necessary step in testing our second hypothesis. The aim here was not an unrealistic one of exactly reproducing the GBM in all respects but to produce a predominantly biological membrane more closely analogous to the *in vivo* GBM and potentially amenable to remodelling by cultured cells. Glomerular cells failed to form monolayers suitable for the proposed model on any of the ECM gels tested. Only when cells were cultured on a rigid collagen membrane (Cellagen) would the cells form a confluent monolayer. This data suggests that not only the composition of the matrix protein, but also its structure and presentation is important in cell monolayer formation, consistent with previous reports [18], [35].

GEnC and podocytes grew in a monolayer (similar to that produced when cells were cultured in tissue culture inserts) on an electrospun collagen I/PCL membrane (Fig. 5). A WST-1 proliferation assay confirmed proliferation of both cell types over 5 days culture on the electrospun collagen I/PCL membrane (Fig. 6). Collagen I/PCL membranes have previously been demonstrated as a suitable material for the culture of endothelial cells to form vascular grafts [36].

In order to allow a thin electrospun nanofibre membrane to be used in the model, it was necessary to provide structural support. Metal scaffolds are regularly used in tissue engineering [37] and nickel is widely used in clinical prostheses because of its inert, non-toxic characteristics. We found that a micro-PEF nickel mesh would be suitable for this application. The thickness of this mesh (and the percentage open area) can be further optimised. We devised and successfully applied a novel approach of electrospinning collagen/PCL nanofibres directly onto the nickel mesh, forming a supported GBM predominantly composed of a biologically remodelable substrate (Fig. 7). Finally we demonstrate the completed model, composed of collagen type I/PCL electrospun onto a nickel mesh, with GEnC growing across this structure in a confluent monolayer, and podocytes growing in a semi-confluent monolayer. The majority of these cells in co-culture are only separated by the electrospun GBM, allowing close contact of the cells and cellular crosstalk.

The composition of the collagen matrix we have developed could be further optimised so that it mimics that of the *in vivo* GBM to a greater extent. For example, one group has already demonstrated that electrospun collagen membranes can be additionally modified by addition of glycosaminoglycan chains [26]. Collagen IV was not studied here because of the lack of availability of the glomerulo-specific isoforms as well as its non-fibrillary nature which does not lend itself to electrospinning. So far there are no publications detailing electrospinning of collagen type IV or laminin-11 and our approach in future development of the model will be to incorporate these proteins into the electrospun collagen type I/PCL nanofibre membranes. This model could also be used to study the effect of different substrates and coculture on cell differentiation. Although we did not see effects on the endothelial and podocyte markers used in this study (PECAM-1 and podocin respectively), study of a panel of such markers including specific matrix component isoforms would be revealing.

In conclusion, we have generated the first tri-layer *in vitro* models of the human glomerular capillary wall containing the relevant cell types, GEnC and podocytes, separated by a membrane and in so doing have successfully confirmed both experimental hypotheses. In the first model, in which this membrane is the porous support of a tissue culture insert, we provide evidence of the cell-cell communication which is important in regulation of the GFB *in vivo*. In the second model, the porous support is replaced with a novel composite bioartificial membrane containing collagen type I, successfully establishing a model where the two cell layers are separated only by a biocompatible membrane over the major of its area. These models will be useful for studying cross-talk between glomerular cells via soluble mediators and the mechanisms of GFB function and how this is disturbed in disease states.

## Materials and Methods

Unless otherwise stated all materials were purchased from Sigma-Aldrich (Poole, UK).

### Cell culture

Human conditionally immortalised GEnC and podocytes were used, as described in detail previously [11], [12]. These cell lines represent the best recognised human culture model of the respective cell types. At the permissive temperature of 33°C, the tsSV40LT transgene is activated, causing cell proliferation, whereas at 37°C, the transgene is inactivated, rendering cells non-proliferative, quiescent and able to differentiate. GEnC and podocyte behaviour was investigated at both 33°C and 37°C. GEnC were cultured in endothelial growth medium 2 microvascular (EGM2-MV, Cambrex, Wokingham, UK) containing FCS (5%) and growth factors as supplied with the exception of VEGF. Podocytes were cultured in RPMI 1640 (Lonza, Basel, Switzerland) as described previously [11]. When cells were grown in co-culture, EGM2-MV media was used.

### Co-culture on tissue culture Inserts

A GEnC or podocyte suspension of 150,000cells/cm^2^ was prepared and 100 µl was pipetted into tissue-culture inserts (1 cm diameter, Nunc Int., Rochester, NY) containing polycarbonate supports (0.4 µm pore size, 0.5 cm^2^ surface area) in 24-well plates. In some cases dual monolayers of the same cell type, or co-cultures of different cell types, were seeded on opposite sides of the membrane. To achieve this inserts were placed upside-down in 24-well plates and 100 µl of cell suspension pipetted onto the support. The cells were then cultured at 33°C for at least four hours to allow cell attachment. The inserts were then turned the correct way up and other cells were pipetted into the insert as above. In all cases total media volume inside and outside the insert was made up to 500 µl. Cells were either cultured for 1 week at 33°C only, or for 1 week at 33°C, then switched to 37°C to differentiate.

### Immunofluorescence

GEnC or podocytes were fixed in 2% paraformaldehyde, blocked and permeabilised with 0.1% Saponin and 3% BSA in PBS and then incubated with an Alexa Fluor 488-phalloidin conjugate (Invitrogen, Paisley, UK) to label the actin cytoskeleton and with DAPI to label nuclei. The polycarbonate supports were removed from their inserts and were mounted on glass slides using Vectashield® aqueous mounting solution (Vector Laboratories, CA). Samples were examined using a cell imaging system (Leica AF6000LX, Leica Microsystems, Wetzlar, Germany).

### Scanning electron microscopy

Samples were fixed in 2.5% glutaraldehyde, dehydrated in graded ethanols. The samples were then dried using a Samdri 780 critical point dryer (Electron Microscopy Sciences, Pennsylvania, USA), then sputter coated with gold using a sputter coater designed at Bristol University. Images were obtained using a Quanta 400FEI scanning electron microscope (FEI, Eindhoven, Netherlands) operated at an accelerating voltage of 15 kV.

### Measurement of transendothelial electrical resistance (TEER)

Cells were seeded in co-culture onto tissue culture inserts as described above. TEER was measured using an Endohm 12 electrode chamber (World Precision Instruments, Sarasota, FL) connected to an EVOMx voltmeter (World Precision Instruments) as previously described [7]. Tissue culture inserts were placed sequentially into the Endohm chamber and resistance recorded after 10 seconds.

### Identification of suitable matrix material

To develop a bioartificial composite membrane we first sought to identify a suitable matrix material. Twelve-well plates were coated with thick matrix gels, before seeding of GEnC at a density of 20,000cells/cm^2^. Gels were made of Collagen type I (Millipore, Billerica, MA), reduced growth factor (RGF) Matrigel (BD Biosciences, San Jose, CA) and Puramatrix peptide hydrogel (BD Biosciences), according to the manufacturers' instructions. In some cases the collagen type I and Matrigel were additionally coated with 20 µg/ml fibronectin or a 2% gelatin solution to enhance cell adhesion. Briefly, 200 µl of solution was pipetted onto the gel and incubated at room temperature for 1 hour. Any excess solution was then removed before cell seeding. Cells were also seeded onto a Cellagen membrane (composed of cross-linked collagen type1, MP Biomedicals, Solon, OH). Cells were cultured for a week at 33°C, then monolayer formation was assessed by light microscopy and images were taken using a CoolPix 4500 camera (Nikon, UK). Cells on Cellagen membranes were visualised by immunofluorescent staining with a VE-cadherin antibody (Santa Cruz Biotechnology, Santa Cruz, CA). Primary antibody binding was detected as above.

### Electrospinning of collagen nanofibres

A 10% collagen solution was prepared by dissolving collagen type 1 from calf skin and 2% PCL (Mn = 80,000) in 1,1,1,3,3,3-hexafluoro-2-propanol (Oakwood, West Columbia, SC, USA). PCL was included as it possesses a high tensile strength, yet is biocompatible and has cell binding properties. The solution was stirred overnight at 37°C.

Electrospinning was performed in a custom-made chamber, where the spinneret was made from a 25 G tip-ground-to-flat needle (Terumo, Hatagaya, Japan), mounted on an electrically insulated stand. The spinneret needle was maintained at a voltage of 12 kV by a high voltage power supply (PS/EL30R01.5-22, Glassman High Voltage Inc., Hampshire UK), and a glass plate covered with a piece of aluminium foil (14×14 cm^2^) under the needle at a distance of 13 cm was grounded and used as the collector. Collagen solutions were spun directly onto the aluminium foil or alternatively across the open lower end of a cylindrical Cell Crown insert (see below) by placing the Cell Crown top down on the foil prior to electrospinning. The capillary needle spinneret was connected through PTFE tubing to a plastic syringe filled with 3 ml of collagen type I spinning solution. A constant volume flow rate of 3 µL/min was maintained using a syringe pump (KD Scientific, MA). Ambient conditions for the current electrospinning process were 49.8% humidity and 21.0°C (room temperature).

Collagen/PCL membranes were cross-linked by immersion in 10%(v/v) HMDI,1,6-diisocyanatohexane (HMDI, Alfa Aesar, MA) in isopropyl alcohol and gently agitated for 2 hours. Membranes were then immersed in isopropyl alcohol for 10 minutes to rinse off HDMI, followed by 1 hour in water.

Membranes were placed into Cell Crowns (Scaffdex, Tampere, Finland) to form an assembly similar to the tissue culture inserts used previously. Cell Crowns consist of 2 polycarbonate rings, one fitting inside the other. The electrospun membrane was placed between the two rings, then one ring was pushed over the other, holding the membrane in place to form an insert. This insert system was then sterilised by exposure to UV light for 30 minutes. The insert was then placed in a 24 well plate. For all experiments membranes were suspended 2 mm above the bottom of the well so that cells could be cultured on either one or both sides of the membrane.

Electrospun membrane stability under tissue culture conditions was tested by placing the membrane in Cell Crowns, then incubating the membrane in culture media at 37°C for 1, 3 and 5 days. At each time point the membrane was examined using light microscopy to demonstrate that fibres remained present and attached to the mesh support.

### Cell culture on electrospun membranes

A GEnC and/or podocyte suspension of 150,000cells/cm^2^ was prepared and 100 µl was pipetted onto the collagen/PCL membrane secured in Cell Crowns. A further 400 µl media was pipetted onto the membrane, and 1 ml media was pipetted outside the insert. In some cases, cells were seeded on both sides as for standard inserts above. Cells were cultured and fixed for immunofluorescence imaging as above. GEnC were incubated with a PECAM-1 antibody (R&D Systems, Minneapolis, MN), whilst podocytes were incubated with a podocin antibody (a podocyte-specific marker, Sigma). The cells were further incubated for 1 hour with an appropriate secondary antibody conjugates (Alexa fluor 488 or 555, Invitrogen), washed, counterstained with DAPI, mounted and imaged as above. When cells were co-cultured, both sides of the membrane were examined and photographed. The cell types were distinguished by staining with different species primary antibodies and secondary antibodies conjugated to different fluorophores.

To confirm that cells maintained viability and proliferation on the collagen/PCL a WST-1 assay (Roche Diagnostics, Mannheim, Germany) was performed 1 and 5 days after seeding. Cells were cultured at 33°C. Metabolically active cells react with tetrazolium salt in WST-1 reagent to produce a soluble formazan dye that can be observed at 450 nm. Prior to this assay, the cellular constructs were rinsed with PBS followed by incubation with media containing 10% WST-1 reagent for 2 hours at 33°C. Thereafter, aliquots were pipetted in triplicate into 96 well plates and the samples read in a Labsystems Multiskan plus colorimetric plate reader at 450 nm (MTX Labsystems, Vienna, VA).

### Nickel mesh support

Micro-PEF nickel mesh with a 19 µm aperture (Tecan, Weymouth, UK) was used as a supportive mesh for the electrospun membrane. Micro-photoelectroforming is a process whereby metal is electrodeposited within a photolithographically defined resist mould. It is a very precise process whereby micro-parts can be manufactured with sub-micron accuracy. This process allows extremely thin meshes to be made. Cells were cultured directly onto this mesh, then stained using antibodies (as previously described) to demonstrate this material was not toxic to cells and that the cells would grow on this material. To produce the complete composite bioartificial membrane, we developed the novel strategy of electrospinning collagen nanofibres directly onto the micro-PEF nickel mesh by using it to replace the aluminium foil in the above process.

### Assembly of the bioartificial model

The bioartificial composite membrane of collagen/PCL electrospun onto nickel mesh was secured in Cell Crowns as described above (Fig. 7C). Cells (GEnC on one side and podocytes on the other) were seeded at a density of 150,000cells/cm^2^. Cells were seeded and incubated for 1 week at 33°C and then switched to 37°C for a week, or differentiated cells were seeded at 37°C.

### Statistical analysis

GraphPad Prism 5 (GraphPad Software, La Jolla, CA) was used for statistical analyses. ANOVA was performed on TEER measurements, and the groups compared using a post hoc Bonferroni multiple comparisons test. A paired Student's t-test was used for WST-1 assay data. P values of <0.05 were taken to indicate statistical significance. Graphs show mean and standard error.
